# Characteristics of Mortality in HIV-negative Cryptococcosis Patients: Analysis of a Cohort of 743 Patients

**DOI:** 10.1093/ofid/ofag410

**Published:** 2026-07-14

**Authors:** Kai Dai, Xinyao Jian, Chongliang Luo, Siguang Wang, Qiyu Pan, Xiaofeng Xu, Jia Liu, Fuhua Peng, Yong Chen, Ying Jiang

**Affiliations:** Department of Neurology, The Third Affiliated Hospital of Sun Yat-sen University, Guangzhou, Guangdong, PR China; Department of Biostatistics, Epidemiology and Informatics, University of Pennsylvania, Philadelphia, Pennsylvania, USA; Division of Public Health Sciences, Washington University School of Medicine in St.Louis, St Louis, Missouri, USA; Department of Neurology, The Third Affiliated Hospital of Sun Yat-sen University, Guangzhou, Guangdong, PR China; Department of Neurology, The Third Affiliated Hospital of Sun Yat-sen University, Guangzhou, Guangdong, PR China; Department of Neurology, The Third Affiliated Hospital of Sun Yat-sen University, Guangzhou, Guangdong, PR China; Department of Neurology, The Third Affiliated Hospital of Sun Yat-sen University, Guangzhou, Guangdong, PR China; Department of Neurology, The Third Affiliated Hospital of Sun Yat-sen University, Guangzhou, Guangdong, PR China; Department of Biostatistics, Epidemiology and Informatics, University of Pennsylvania, Philadelphia, Pennsylvania, USA; Department of Neurology, The Third Affiliated Hospital of Sun Yat-sen University, Guangzhou, Guangdong, PR China

**Keywords:** cryptococcal meningitis, cryptococcosis, HIV-negative, mortality, risk factors

## Abstract

**Background:**

Reports of cryptococcosis among patients without human immunodeficiency virus (HIV) infection are increasing. However, there is still a lack of data regarding the mortality characteristics in this population.

**Method:**

The clinical, laboratory, imaging data from 743 HIV-negative patients with cryptococcosis were analyzed. Kaplan–Meier analysis was used to estimate all-cause mortality at 2-week, 10-week, and 1-year, with comparisons between groups using log-rank tests. Risk factor analysis was conducted using Cox models. Additionally, a meta-analysis was conducted on four recent large-scale cohort studies to investigate the differences in 2-week mortality of HIV-negative cryptococcosis across different regions. Statistical analyses were performed using R.

**Result:**

The 2-week, 10-week, and 1-year mortality rates were 1.4% (95% confidence interval [CI] 0.5–2.2%), 8.4% (95% CI 6.3–10.4%), and 12.2% (95% CI 9.7–14.7%), respectively. Old age, higher baseline modified Rankin Scale scores, altered mental status, elevated total bilirubin levels, increased peripheral white blood cell counts, reduced serum albumin levels, elevated creatinine levels were associated with the mortality of cryptococcosis patients. For investigating the difference in 2-week mortality between Western and Chinese HIV-negative cryptococcosis, meta-analysis identified solid organ transplant and malignancy as risk factors (RR = 1.50, 95% CI 1.08–2.07, *P* = .02), while meta-regression indicated that older age in HIV-negative patients in Western countries increased risk (RR = 1.15, 95% CI 1.09–1.22, *P* < .001).

**Conclusions:**

The mortality rate of HIV-negative cryptococcosis patients is relatively low and primarily occurs within 2 to 10 weeks. The characteristics contributing to a lower 2-week mortality rate differ significantly from those in the Western countries, mainly because of the differences in age and underlying risk populations.

Cryptococcosis is a globally significant infection caused by the pathogenic yeast *Cryptococcus*. It primarily infects the lungs through inhalation and spreads to various parts of the body, including the central nervous system (CNS), skin, prostate, eyes, bones, and joints [1–3]. The global burden and mortality rate associated with cryptococcosis remain high [4]. In recent years, with the widespread use of highly effective antiretroviral therapy, the incidence of cryptococcosis among HIV-positive patients has decreased [4, 5]. However, there is an increasing number of reports of *Cryptococcus* infections among HIV-negative patients [5, 6], and these patients have now become a significant component of the global cryptococcal disease burden.

Most investigations of mortality in cryptococcosis concentrate on HIV-positive patients, especially those diagnosed with cryptococcal meningitis (CM). The short-term mortality rate for HIV-positive cryptococcal infection ranges from 17% to 26% at 2-week and from 15% to 53% at 10-week [6–12]. The 1-year mortality rate for HIV-positive cryptococcal infection ranges from 22% to 78% [5, 6, 9–13]. However, studies on HIV-negative cryptococcosis are relatively scarce, which is often limited by the small number of patients or the lack of detailed clinical data [5, 13, 14]. The short-term mortality rates for HIV-negative cryptococcal infection are relatively lower, with 2.7% to 17.6% at 2-week [15, 16], 11.5% to 12.2% at 10-week [5, 7] and 13% to 42% at 1-year [5, 6, 13, 15]. Previous study has indicated that there are notable differences in mortality rates among different subgroups of HIV-negative patients with cryptococcal infection, including subgroups based on patient characteristics, *Cryptococcus* species, and infection sites [5]. Due to the heterogeneity of HIV-negative individuals and the lack of large sample data, the evidence regarding mortality characteristics in this population still remains insufficient.

Currently, the significant regional disparities exist in global data on HIV-negative cryptococcosis. Most evidence originates from Western countries [5, 16], while data from high HIV-burden regions-such as African Region and South-East Asia Region-are lacking [8, 9]. In these regions, limited diagnostic capacity and inadequate healthcare resources may lead to underdiagnosis of HIV-negative cryptococcosis, thereby masking its true mortality rates and clinical characteristics [4, 12]. Similarly, the data in China are also rare. To fill this gap, we conducted a single-center retrospective study of the largest population with HIV-negative cryptococcosis in the world, aiming to update the characteristics of mortality in HIV-negative patients.

## MATERIALS AND METHODS

### Ethics Statement

This study was conducted following the principles of the Helsinki Declaration and approved by the Medical Ethics Committee of the Third Affiliated Hospital of Sun Yat-sen University (Ethics No. 2025-233-01). Due to the retrospective design of this study and the use of fully anonymized patient medical records, the requirement for written informed patient consent was waived by the institutional ethics committee. This study does not involve any additional factors that require patient consent.

### Study Design

This study was conducted following the principles of the Helsinki Declaration and approved by the Medical Ethics Committee of the Third Affiliated Hospital of Sun Yat-sen University (Ethics No. 2025-233-01). We retrospectively analyzed the medical records of 790 Chinese Han HIV-negative cryptococcosis patients admitted to the Third Affiliated Hospital of Sun Yat-sen University, Guangzhou, Southern China from 1 January 2011, to 31 December 2022. Patient records were retrieved from databases, including microbiological and histopathological information, and screened for inclusion criteria. Among them, 743 cryptococcosis patients (94.1%) were included in this study. Detailed inclusion/exclusion criteria and the patient enrollment flowchart are provided in the Supplementary Figure 1.

### Definitions

The definition of cryptococcosis was based on the consensus definition of invasive fungal diseases by the European Organization for Research and Treatment of Cancer (EORTC) and the Mycoses Study Group Education and Research Consortium (MSG-ERC), revised and updated in 2019 [17]. Confirmed cases were those diagnosed by microscopy, culture, antigen, and tissue nucleic acid detection [17]. Disseminated cryptococcosis is defined as infection involving at least two noncontiguous sites. As most of the cryptococcosis patients in this study did not undergo blood culture testing, we were unable to determine whether they had fungemia. Therefore, all patients with CNS cryptococcal infections, regardless of the presence of fungemia, were classified as CNS cryptococcosis. Persistent infection was defined as a persistently positive cryptococcal culture in cerebrospinal fluid (CSF) for 4 weeks after starting antifungal therapy [18]. Microbiological relapse was defined as recurrence of symptoms, with recovery of viable organisms from previously sterile CSF [18]. Paradoxical immune inflammatory response syndrome (PIIRS) is defined as paradoxical clinical and/or radiological deterioration in CM patients who were previously healthy and without immunological abnormalities, occurring during effective antifungal treatment due to a strong immune response, with consistently negative fungal cultures [19, 20]. Altered mental status was defined as a Glasgow coma score (GCS) of <15. Detailed definitions are in Supplementary Statistical Analysis.

### Data Collection

All patients' demographic and clinical data were recorded in standardized forms. We collected demographic and clinical characteristics of patients with cryptococcosis at admission, including age, gender, residence, underlying diseases, onset symptoms, time from symptoms to diagnosis, laboratory investigations, cranial radiology, lumbar puncture (LP) results, serum and CSF CrAg test results, antifungal treatment, and outcomes (eg, death status). After discharge, patients were followed up either at outpatient visits or via telephone interview. Accurate outcome information for patients was documented at 2-week, 10-week, and 1-year post-discharge. Patients who cannot be followed up for 1 year were considered lost to follow-up. For these lost-to-follow-up patients, the final follow-up time point and outcome information were recorded. For the meta-analysis, a PubMed search found 104 records, and 3 studies were included after applying criteria.

### Statistical Analysis

The Shapiro–Wilk test checked for normality in continuous variables. Non-normal variables were reported as medians with interquartile ranges, and normal variables as means with standard errors. Group comparisons used the Kruskal–Wallis test for non-normal data and one-way ANOVA for normal data. Categorical variables were compared using the Chi-squared test, with Fisher's exact test for variables with fewer than five groups. Kaplan–Meier analysis estimated overall mortality, and log-rank tests compared groups. Hazard ratios and confidence intervals were calculated using Cox models. A mortality index score, based on the Cox model-derived weighted sum of risk factors, was compared between patient groups. The meta-analysis contains 4 studies (including ours) that analyzed the association between risk factors and 2-week mortality in HIV-negative cryptococcosis patients. The meta-analysis was conducted using random-effect model and visualized by a forest plot. A further meta-regression analysis via mixed-effects Poisson regression was conducted to evaluate the effect of age on 2-week mortality. All analyses were done using R. Detailed methods are in Supplementary Statistical Methods.

## RESULTS

### Socio-demographic Characteristics

Characteristics of the 743 HIV-negative patients with cryptococcosis are shown in Table 1 and Supplementary Table 1. The median age of the patients was 47 years (IQR 37–59). Among them, 68.8% (511/743) were males, with a male-to-female ratio of 2.2:1 (511/232).

**Table 1.** Characteristics of the 743 HIV-Negative Patients With cryptococcosis

Characteristic	All Patients (*N* = 743)	Immune Condition		*P*-value
		Patients with at least one known underlying immunocompromising condition(s) before cryptococcosis (N = 304)	Patients with no identifiable immunocompromising condition (N = 439)	
Male	511 (68.8)	192 (63.2)	319 (72.7)	.01
Age, y, median (IQR)	47.0 (37.0, 59.0)	47.5 (38.0, 59.0)	47.0 (36.0, 59.0)	.66
Initial mRS score, mean ± SD	1.0 (1.0, 2.0)	1.0 (1.0, 2.0)	1.0 (1.0, 2.5)	.35
Initial GCS score <15	96 (12.9)	39 (12.8)	57 (13.0)	1
Site (s) of infection				…
CNS	676 (91.0)	279 (91.8)	397 (90.4)	.62
Lung	78 (10.5)	30 (9.9)	48 (10.9)	.73
Blood^a^, *n* = 289	34 (11.8)	19 (15.3)	15 (9.1)	.15
Skin	5 (0.7)	2 (0.7)	3 (0.7)	1
Bone	4 (0.5)	2 (0.7)	2 (0.5)	1
Other (soft tissue, adrenal gland, and/or prostate)	2 (0.3)	0 (0.0)	2 (0.5)	.52
Disseminated	51 (6.9)	26 (8.6)	25 (5.7)	.17
If symptomatic: time from onset of symptoms to diagnosis, d, median (IQR), *n* = 702	23.5 (14.0, 49.0)	22.0 (12.0, 42.2)	25.0 (14.2, 53.0)	.03
Cryptococcus species, *n* = 257				…
*C. neoformans*	239 (93.0)	122 (97.6)	117 (88.6)	.01
*C. gattii*	18 (7.0)	3 (2.4)	15 (11.4)	.01
Baseline blood chemistry, median (range)				…
Aspartate aminotransferase, U/L, *n* = 713	20.0 (15.0, 29.0)	21.0 (15.0, 29.8)	19.0 (15.0, 29.0)	.37
Alanine aminotransferase, U/L, *n* = 708	26.0 (16.0, 43.0)	26.0 (15.2, 40.0)	26.0 (16.0, 45.8)	.32
Total protein, g/L, *n* = 706	65.6 (60.4, 70.7)	63.4 (57.4, 68.9)	66.7 (62.7, 72.3)	<.01
Albumin, g/L, *n* = 707	39.5 (35.8, 42.6)	37.5 (33.3, 41.2)	40.6 (37.5, 43.5)	<.01
Total bilirubin, µmol/L, *n* = 617	9.7 (6.4, 14.3)	10.2 (6.3, 15.7)	9.6 (6.4, 13.9)	.25
Sodium, mmol/L, *n* = 710	137.0 (133.0, 140.1)	137.0 (133.0, 140.0)	136.6 (133.0, 140.3)	.83
Chloride, mmol/L, *n* = 710	98.1 (94.5, 102.6)	98.7 (95.1, 103.0)	97.8 (94.1, 102.0)	.08
Blood urea nitrogen, mmol/L, *n* = 365	4.7 (3.5, 6.3)	4.7 (3.3, 6.0)	4.7 (3.6, 6.4)	.37
Creatinine, µmol/L, *n* = 715	67.0 (54.0, 85.0)	68.0 (54.0, 88.0)	66.0 (54.0, 83.0)	.29
C-reactive protein, mg/L, *n* = 381	6.9 (2.2, 20.6)	8.6 (2.5, 23.5)	6.0 (2.1, 18.4)	.12
Baseline bloodwork, median (range)				…
White blood cell, 10^9^/L, *n* = 722	8.4 (6.2, 11.6)	7.7 (5.7, 10.6)	8.9 (6.6, 12.0)	<.01
Hemoglobin, g/L, *n* = 725	125.0 (110.0, 139.0)	121.5 (105.0, 136.0)	127.0 (114.0, 141.0)	<.01
Platelet, g/L, *n* = 726	247.0 (191.2, 305.0)	226.0 (157.0, 277.0)	259.0 (219.0, 319.0)	<.01
CSF findings (for patients with CNS cryptococcosis)				
Opening pressure, cmH_2_O, *n* = 652				
<20	216 (33.1)	95 (35.1)	121 (31.8)	.28
20–30	279 (42.8)	106 (39.1)	173 (45.4)	.28
>30	157 (24.1)	70 (25.8)	87 (22.8)	.28
CSF cryptococcus count, count/mL, median (IQR), *n* = 567	1320.0 (25.0, 12297.0)	948.5 (22.0, 12682.0)	1560.0 (36.0, 11246.0)	.66
White blood cell, 10^6/L, median (IQR), *n* = 668	82.0 (32.0, 174.0)	78.0 (28.0, 172.0)	87.0 (34.0, 174.5)	.27
Glucose, mmol/L, median (IQR), *n* = 664	0.8 (0.5, 1.2)	0.7 (0.5, 1.2)	0.8 (0.5, 1.3)	.12
Protein, g/L, median (IQR), *n* = 664	1.8 (0.8, 2.7)	2.1 (0.9, 2.9)	1.7 (0.8, 2.5)	<.01
Chloride, mmol/L, median (IQR), *n* = 664	117.5 (112.1, 121.9)	117.5 (112.2, 121.9)	117.5 (111.9, 121.9)	.88
Positive India ink staining, *n* = 674	591 (87.7)	240 (86.3)	351 (88.6)	.44
Positive culture, *n* = 665	424 (63.8)	196 (71.0)	228 (58.6)	<.01
Positive CSF CrAg, *n* = 260	258 (99.2)	126 (99.2)	132 (99.2)	1
Positive serum CrAg, *n* = 189	180 (95.2)	90 (94.7)	90 (95.7)	1
Positive blood cultures, *n* = 289	34 (11.8)	19 (15.3)	15 (9.1)	.15
All-cause mortality, 95% CI				…
2 wk	1.4 (0.5, 2.19)	1.0 (0.0, 2.1)	1.59 (0.4, 2.8)	.48
10 wk	8.4(6.3–10.4)	10.53 (7.5, 14.9)	6.2 (4.1, 8.8)	.03
1 y	12.2(9.7–14.7)	13.5 (10.4, 19.0)	9.8 (7.5, 13.6)	.1

Data are *n* (%) unless otherwise indicated. a: means the result of positive Cryptococcus in blood culture. b: specific medications and surgical treatments are detailed in Supplementary Table 4. CrAg testing was implemented at our institution in 2018.

Abbreviations: IQR, interquartile range; mRS, modified Rankin Scale; GCS, Glasgow Coma Scale; CNS, central nervous system; CSF, cerebrospinal fluid; CrAg, cryptococcal antigen; CI, confidence interval; HIV, human immunodeficiency virus; PIIRS, paradoxical immune inflammatory response syndrome; IRIS, immune reconstitution inflammatory syndrome.

### Clinical Characteristics

Clinical characteristics of cryptococcosis are presented in Table 1 and Supplementary Table 1. The common sites of infection were the CNS (91.0%, 676/743), lungs (10.5%, 78/743), followed by skin (0.7%, 5/743), bones (0.5%, 4/743), and other sites (0.3%, 2/743). Blood infections were documented in 11.8% (34/289) of cases. The most frequently reported clinical manifestations were neurological symptoms. Headache was the predominant symptom, present in 82.4% of patients, followed by other neurological features (88.7%). Systemic symptoms were also common, with fever (≥38℃) observed in 41.3% of the cohort. Nausea or vomiting, were reported in 44.0% of patients. The prevalence of these common symptoms was largely consistent between patients with and without an identifiable immunocompromising condition. In symptomatic patients, the median time from symptom onset to diagnosis was 23.5 days (IQR 14 to 49). Only 3.5% (26/743) of cryptococcosis patients were asymptomatic, and all of these patients (26/26) were only pulmonary cryptococcosis. Moreover, 40.9% (304/743) of patients had at least one underlying immunocompromising condition (see Supplementary Table 2).

### Diagnostic/Investigation Results

The clinical microbiology findings, other laboratory pathological results, and imaging findings are detailed in Table 1. Blood cultures were performed for 38.0% (282/743) of all the patients, among which 11.8% (34/282) were tested positive for *Cryptococcus*. In addition, CrAg testing (serum and CSF) was first performed in 2018. Serum cryptococcal antigen (CrAg) testing was conducted on 25.4% (189/743) of cryptococcosis patients, among which 95% (180/189) yielded positive results. A total of 9 patients in our cohort presented negative serum CrAg results. Among them, 3 patients were diagnosed with pulmonary cryptococcosis, which was confirmed by lung histopathological biopsy. The remaining 6 patients were diagnosed with CNS cryptococcosis. Of these 6 cases, 3 were confirmed by positive CSF fungal culture, and the other 3 were confirmed by India ink staining. Among CNS cryptococcosis, the positivity rates for CSF India ink staining and cryptococcal culture were 87.7% (591/674) and 63.8% (424/665), respectively. *Cryptococcus* species identification was performed on CSF samples from 257 patients. Among 257 patients, 93% (239/257) were identified as *C. neoformans*, and 7% (18/257) were identified as *C. gattii*. Moreover, 38.3% (259/676) of CNS cryptococcosis were tested for CSF CrAg, of which all (100%, 259/259) were tested positive. Baseline blood tests are detailed in Table 1. In the brain MRI results of 588 CNS cryptococcosis patients, common manifestations include meningeal enhancement (48.5%, 285/588) and hydrocephalus (18%, 106/588), followed by cryptococcoma(s) (15.0%, 88/588) and pseudocysts (1.7%, 10/588). However, 38.4% (226/588) of CNS infection patients did not exhibit characteristic lesions.

### Treatment Protocols

The treatment regimens are detailed in Table 2. Among 676 patients with CNS cryptococcosis, 96.7% (654/676) received induction treatment lasting more than 2 weeks. Of these, 56.6% (370/654) received initial therapy based on amphotericin B (AmB). For 62 patients with pulmonary cryptococcosis, 100% (62/62) were treated with FLU monotherapy.

**Table 2.** Therapy of the 743 HIV-Negative Patients With Cryptococcosis

Treatment	
CNS cryptococcosis, *n* = 654	
AmB-based initial therapy	370 (56.6)
AmB+FLU+5-FC	197 (30.1)
AmB+VOR + 5-FC	123 (18.8)
AmB+5-FC	50 (7.6)
FLU-based initial therapy^A^	254 (38.8)
FLU+5-FC	34 (5.2)
FLU	220 (33.6)
Others	30 (4.6)
Pulmonary cryptococcosis, *n* = 62	
FLU	62 (100.0)
Surgical treatment ^B^(for patients with CNS cryptococcosis), *n* = 171	
Ventriculoperitoneal shunt	161 (94.2)
Extra ventricular drainage	9 (5.3)
External drainage of lumbar cistern	3 (1.8)
Implantation of Omaya's capsule	2 (1.2)
Ventriculoperitoneal shunt combined with external ventricular drainage	2 (1.2)
Ventriculoperitoneal shunt combined with external drainage of lumbar cistern	1 (0.6)
External drainage of lumbar cistern combined with implantation of Omaya's capsule	1 (0.6)

Data are *n* (%) unless otherwise indicated. A: The relatively high proportion of fluconazole monotherapy (220 cases, 33.6%) can be attributed to two main reasons: firstly, patients opting for non-AmB-based regimens often declined AmB due to concerns about its side effects (particularly nephrotoxicity), financial considerations, and their underlying comorbidities (it should be noted that liposomal amphotericin B was not available in our hospital until June 2022); secondly, the treatment of cryptococcosis in our institution was mainly referenced to the 2010 IDSA guidelines and the 2018 expert consensus on the diagnosis and management of cryptococcal meningitis issued by the Chinese Society of Infectious Diseases. However, the standardized treatment protocols were not fully established in the earlier years of this retrospective period, and individual clinical physicians had variations in their therapeutic experience, leading to some variability in clinical treatment choices in this study. In recent years, the majority of patients with CNS cryptococcosis have received the amphotericin B (AmB)-based therapy. In addition, the dosage of fluconazole was 800 mg once daily. B: VP shunt indications: VP shunt may be performed when clinically necessary if conservative drainage fails after standardized antifungal therapy, without awaiting complete control of CNS infection. Early VP shunt is recommended for patients with persistently elevated ICP > 300 mmH_2_O, particularly those complicated by cranial nerve impairment, visual or auditory dysfunction, or altered consciousness. Early VP shunt is strongly advised for patients with a GCS score < 15, which also accounts for the high proportion of VP shunt procedures in this study.

Abbreviations: CNS, central nervous system; AmB, amphotericin B; FLU, fluconazole; 5-FC, 5-flucytosine; VOR, voriconazole; HIV, human immunodeficiency virus.

Among the 676 patients with CNS cryptococcosis, a total of 30.3% (205/676) of patients underwent surgical intervention to manage intracranial pressure. Among them, 83.4% (171/205) received surgery within 2 weeks of diagnosis. The surgical methods are detailed in Table 2.

### Outcomes

#### Mortality rate

At the 2-week, complete data were available for 728 patients (98.0%), with 15 patients lost to follow-up. The overall 2-week mortality rate was 1.4% (95% CI .5–2.2%). At the 10-week, complete data were available for 687 patients (92.5%), with 56 patients lost to follow-up. The overall 10-week mortality rate was 8.4% (95% CI 6.3–10.4%). At the 1-year, complete data were available for 648 patients (87.2%), with 95 patients lost to follow-up. The overall 1-year mortality rate was 12.2% (95% CI 9.7–14.7%).

**Figure 1.**
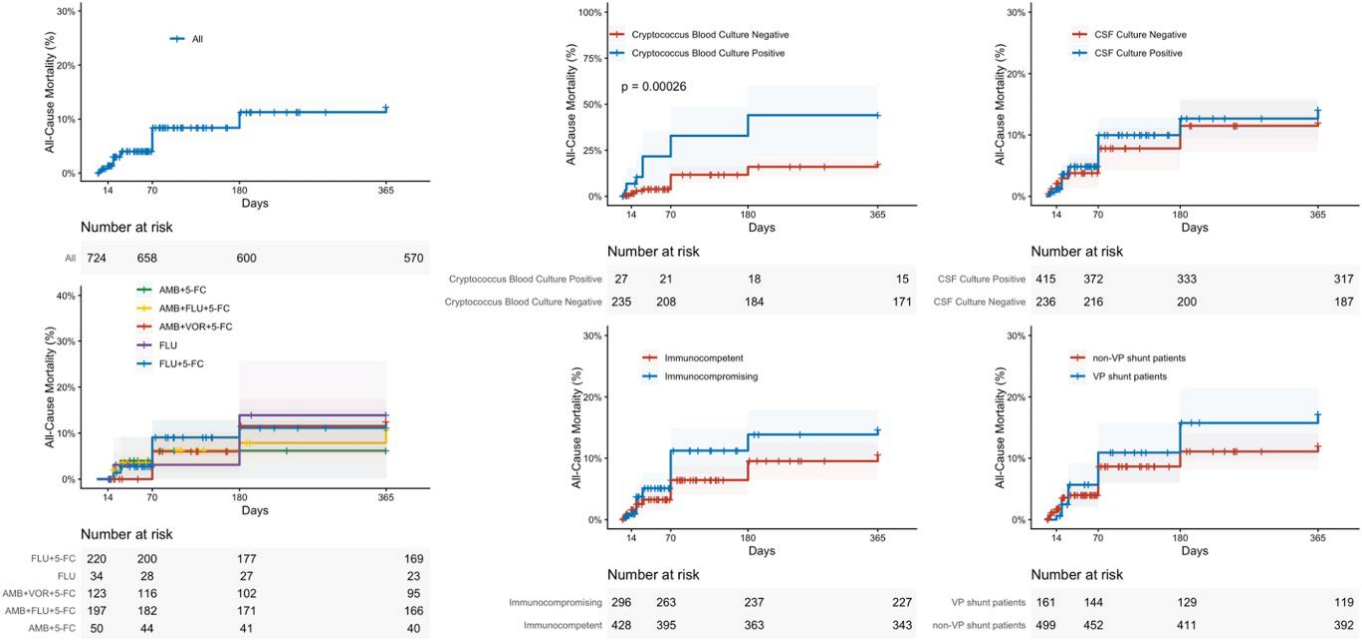
Analysis of cumulative all-cause mortality at 1-y for the overall patient population and different subgroups. (A) All cryptococcosis patients. (B) Blood culture. (C) CSF culture. (D) Medication treatment regimens. (E) Immune function. (F) Surgical treatment.

In the subgroup analysis, Kaplan–Meier survival analysis revealed no significant differences across all comparisons for 2-week mortality rate (see Figure 1). Patients with cryptococcal positive blood cultures had a significantly higher 10-week mortality rate of 30.0% (95% CI 12.7%–48.4%) compared to those with cryptococcal negative blood cultures, whose 10-week mortality rate was 10.8% (95% CI 7.4%–15.9%) (Log-rank *P* < .001) (see Figure 1). Furthermore, patients with positive cryptococcal blood cultures had a significantly higher 1-year mortality rate (40.0%, 95% CI 21.8%–60.0%) compared to those with negative blood cultures (15.8%, 95% CI 12.3%–22.4%) (Log-rank *P* < .001) (see Figure 1).

#### Risk factors for mortality and mortality index scores

**Figure 2.**
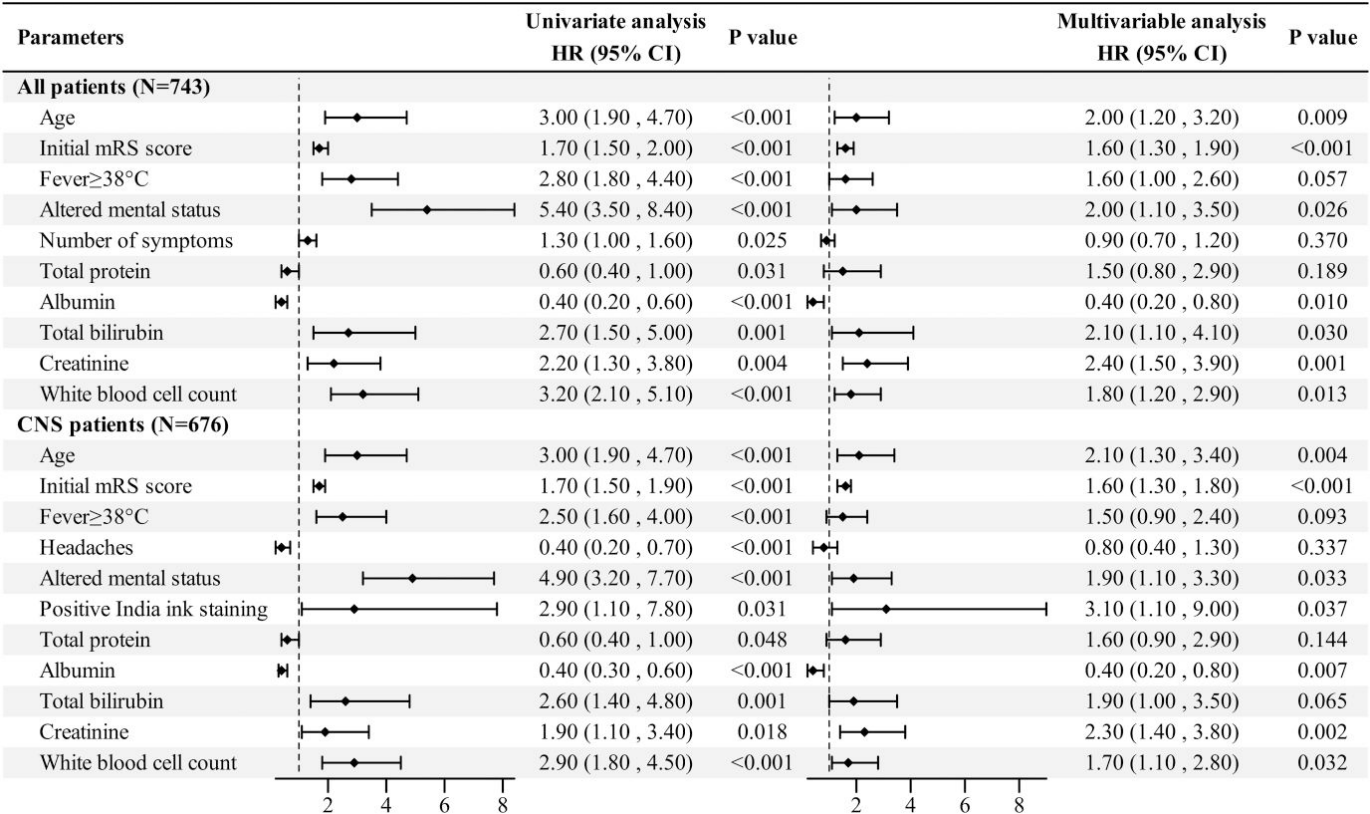
Forest plot of the results of the COX proportional hazards regression model for patients with overall cryptococcosis.

The multivariable Cox regression model identified age, initial mRS score, altered mental status, ALB, TBIL, creatinine, and peripheral WBC factors with significant adjusted hazard ratios for overall cryptococcosis and CNS cryptococcosis patients respectively (see Supplementary Table 2 and Figure 2). Among HIV-negative cryptococcosis patients, age (HR 2.0, 95% CI 1.2–3.2, *P* = .009), initial mRS scores (HR 1.6, 95% CI 1.3–1.9, *P* < .001), altered mental status (HR 2.0, 95% CI 1.1–3.5, *P* = .03), ALB (HR 0.4, 95% CI .2–.8, *P* = .01), TBIL (HR 2.1, 95% CI 1.1–4.1, *P* = .03), creatinine (HR 2.4, 95% CI 1.5–3.9, *P* = .001), and increased peripheral WBC count (HR 1.8, 95% CI 1.2–2.9, *P* = .01) were associated with mortality. Similar risk patterns emerged in CNS cases, and an additional risk factor was positive India ink staining (HR 3.1, 95% CI 1.1–9.0, *P* = .04).

**Figure 3.**
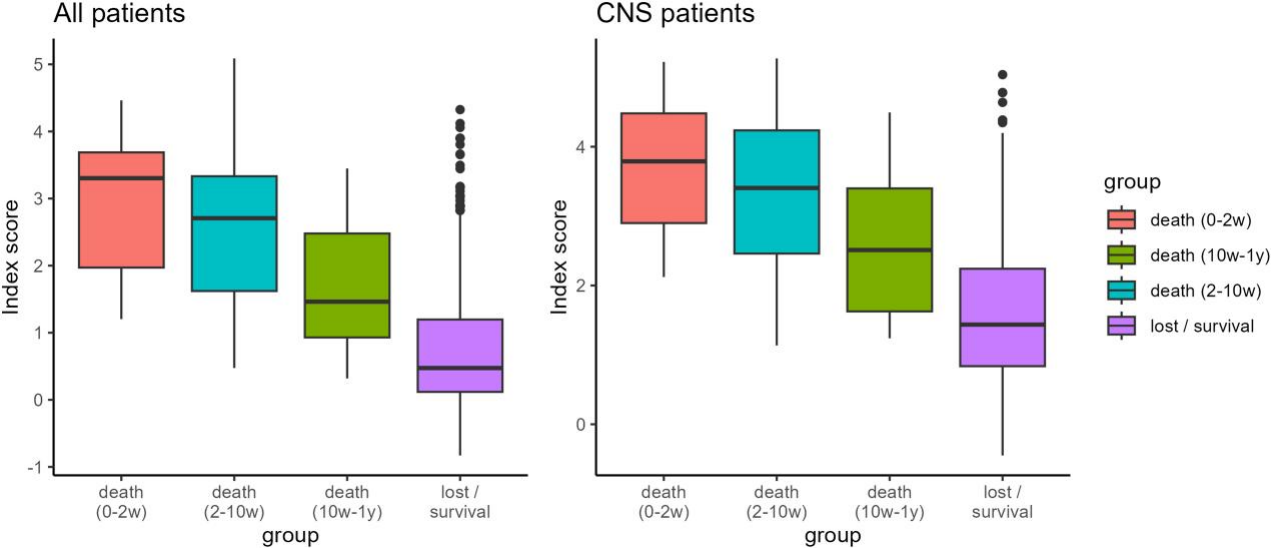
The mortality index scores by the death time groups. The index scores were derived as the weighted sum of significant risk factors from the multivariable Cox models. Left: all patients; right: CNS patients.

The mortality index scores derived from the Cox models are presented in Figure 3, where the statistical significance of the differences between groups using *t* tests are: *P* = .368 for death 0–2 weeks versus 2–10 weeks, *P* = .001 for death 2–10 weeks versus 10 week–1 year, and *P* < .001 for death 10 weeks–1 year versus lost follow-up or survived for all patients, and *P* = .437 for death 0–2 weeks versus 2–10 weeks, *P* = .004 for death 2–10 weeks versus 10 weeks–1 year, and *P* < .001 for death 10 weeks–1 year versus lost follow-up or survived for CNS patients.

#### Meta-analysis of different conditions and 2-week mortality

The meta-analysis contained 4 studies (including ours) to analyze the difference in 2-week mortality between patients with solid organ transplant (SOT)/malignancy and other conditions. The data are shown in Table 3 and Supplementary Table 3. The random-effects meta-analysis model indicated that SOT and malignancy, compared to other or no immunocompromising conditions, has higher risk of 2-week mortality, with a summarized RR = 1.50 (95% CI: 1.08–2.07, *P* = .02, heterogeneity *I*^2^ = 0, *P* = .53) (see Supplementary Figure 2). Further meta-regression analysis showed that older age is associated with the increased 2-week mortality in the Western studies, with an estimated RR as 1.15 per 1-year older average age, (95% CI = 1.09–1.22, *P* < .001).

**Table 3.** Comparison of the Basic Characteristics and 2-Week Mortality in Studies on Cryptococcosis in HIV-Negative Patients

Characteristics	2-wk Mortality			
	Australia and New Zealand	France	Eastern China	Our study
All patients	30/426 (7.0%)	96/547 (17.6%)	16/584 (2.7%)	10/743 (1.3%)
Underlying risks				
Malignancy	5/82 (6.4%)	43/177 (24.3%)	0/30 (0%)	0/25 (0%)
Solid organ transplant	12/81 (14.5%)	16/114 (14%)	0/15 (0%)	0/13 (0%)
Other immunocompromising condition	3/96 (3.2%)	28/174 (16.1%)	10/238 (4.2%)	3/266 (1.1%)
No identified condition	10/167 (6.1%)	9/82 (11%)	6/301 (2.0%)	7/439 (1.6%)
Cryptococcus species				
*C. gatti*	0/82 (0%)	NA	NA	0/18 (0%)
*C. neoformans*	28/228 (12.3%)	NA	NA	2/239 (0.9%)
Site(s) of infection				
CNS involvement	20/195 (10%)	NA	16/584 (2.7%)	10/676 (1.5%)
No CNS involvement	12/231 (5%)	NA	NA	0/67 (0%)

Abbreviation: NA, not available.

Australia and New Zealand, France, Eastern China: Data from these regions were obtained from previously published studies [5, 15, 16]. In the Australian study, the 2-wk mortality rate was indirectly obtained based on the survival curves provided in the Supplementary materials. Our study: Data from our study.

## DISCUSSION

This study represents the largest cohort to date comprehensively characterizing mortality patterns in HIV-negative cryptococcosis patients. Our analysis yielded several key findings: First, the overall mortality rates were 1.4% at 2 weeks, 8.4% at 10 weeks, and 12.2% at 1 year, confirming a substantially lower burden than in HIV-positive populations. Second, we identified a distinctive temporal mortality pattern, with deaths occurring predominantly between 2 and 10 weeks after diagnosis, contrasting sharply with the early mortality peak observed in Western cohorts. Third, through meta-analysis, we demonstrated that the marked discrepancy in 2-week mortality between Chinese and Western patients (1.3–2.7% vs 7.0–17.6%) is primarily attributable to differences in age distribution and underlying risk conditions—specifically, the higher proportion of elderly patients with solid organ transplantation or malignancy in Western populations. Fourth, we established a practical risk stratification model incorporating readily available clinical parameters (age, initial mRS, mental status, albumin, total bilirubin, creatinine, and white blood cell count) that effectively discriminates mortality risk across different time horizons. These findings collectively advance our understanding of HIV-negative cryptococcosis as a distinct entity with region-specific mortality determinants and provide actionable insights for targeted management strategies.

The mortality rate for HIV-positive cryptococcosis ranged from 17% to 26% at 2-week, from 15% to 53% at 10-week, and the 1-year mortality rate ranged from 22% to 78% [5, 6, 9–13]. We have clarified that even within the same region and under a similar healthcare system in South China, the 1-year mortality rate of HIV-positive cryptococcosis patients remains above 30% [21]. In this study, the 2-week mortality rate for overall cryptococcosis was 1.4%, the 10-week mortality rate was 7.9%, and the 1-year mortality rate was 12.2%. Compared to the high mortality rate in HIV-positive patients [5–13], both the short-term and long-term mortality rates in HIV-negative cryptococcosis patients appear to be relatively lower. Notably, the mortality rate of HIV-positive patients remains consistently high during both the 0–2 weeks and 2–10 weeks periods [8, 11, 12]. In contrast, our study shows that deaths of HIV-negative patients mainly occurred between 2 and 10 weeks. This finding is consistent with previous studies in China [7, 15], indicating that HIV-negative patients from the China have a relatively lower mortality rate in the early stages of the disease (0–2 weeks), but it significantly increases in the subsequent period (2–10 weeks). Unfortunately, the cause of this delayed mortality pattern remains unexplored.

The delayed mortality pattern observed in our cohort—with the minimal number of early deaths followed by an upward trend within 2–10 weeks—represents a clinically significant novel observation. Recent two large studies from the Western countries [5, 16] showed that HIV-negative cryptococcosis patients had a relatively high 2-week mortality rate, ranging from 7.0% to 17.6%. Our meta-analysis and meta-regression definitively establish that this difference reflects fundamental population differences: Western HIV-negative cryptococcosis predominantly affects older individuals with SOT or malignancy, populations inherently vulnerable to early mortality, whereas Chinese patients are typically middle-aged (median 47 years) with no identifiable immunocompromising condition (59.1%). Moreover, a growing number of epidemiological studies have provided evidence that age and the high-risk populations are independently associated with an increased risk of 2-week mortality in HIV-negative cryptococcosis patients [22, 23]. This finding challenges the prevailing paradigm, derived largely from Western studies, that HIV-negative cryptococcosis carries substantial short-term mortality risk. Instead, our data indicate that outcomes are highly dependent on specific circumstances and vary depending on regional epidemiological profiles. For regions dominated by young-to-middle-aged immunocompetent hosts, early mortality may be considerably lower than current estimates. This observation has direct clinical relevance. The risk stratification tools developed in Western populations may overestimate early risk when applied to Asian populations, while interventions proven effective in Western cohorts may not yield the same benefit in settings with already low early mortality rates. Future research should focus on developing region-specific prognostic algorithms and investigating whether the delayed mortality window in Asian patients provides therapeutic opportunities for interventions targeting inflammatory complications or enhanced fungal clearance.

Consistent with previous findings in studies on HIV-negative patients [6, 15, 24, 25], independent predictors with cryptococcosis patients included reduced ALB levels, elevated peripheral WBC counts, altered mental status and older age. Additionally, we found that the higher initial mRS score and elevated TBIL levels were predictors of mortality. A higher initial mRS score indicates more severe neurological deficits and self-care limitations before the disease. Elevated TBIL level may suggest potential liver dysfunction, which could negatively impact the overall prognosis. For patients with CNS cryptococcosis, similar to overall cryptococcosis patients, an additional factor is positive India ink staining. Positive India ink staining at baseline generally indicates a higher fungal burden at the initial stage, which is closely associated with a significant mortality rate in HIV-negative patients [15]. The mortality index scores derived from the Cox models also shows good separation of the patient risk groups and is potentially useful for early prediction or stratification of the patient mortality risk at the baseline. Interestingly, similar to a previous study on HIV-negative CM in China [24], pharmacological treatment may not be the primary factor influencing the survival of HIV-negative patients. However, pulmonary cryptococcosis treated with FLU monotherapy has a good prognosis based on our data. Therefore, further study is needed to explore the complex relationship between drug efficacy and prognosis.

Similar to previous reports indicating that cryptococcemia accounts for only 10% to 30% of all cryptococcal diseases [26], our study found that 11.8% (34/282) of patients had cryptococcemia. Notably, 97.1% (33/34) of the cryptococcemia patients in our study had CNS involvement, which was higher than 71.0% to 89.3% reported in previous literature [27]. Prior studies have reported a 30-day mortality rate for cryptococcemia ranged from 35% to 54.1% [26, 27], while our study found a 10-week mortality rate of these patients was as high as 30%. Cryptococcemia reflects a higher fungal burden in patients, indicating more severe disease and increased difficulty in treatment [28], thereby increasing the risk of mortality in cryptococcosis patients [7]. We recommend routine blood cultures and LP examinations for patients with cryptococcal infections to facilitate early identification of cryptococcemia and involvement of CNS, so as to better treat and manage these patients.

There are several limitations in this study. First, the data were sourced from a single center, which may limit the generalizability of the results. Second, due to the retrospective study design, some patients were lost to follow-up at different time points, which may affect the study outcomes. Additionally, some variables, such as blood culture results, *Cryptococcus* species identification and CrAg test, were incomplete, which data may have led to bias in the calculation of mortality rates in this study. Diagnostic and antifungal therapeutic strategies exhibited temporal heterogeneity over the study period. CrAg testing and formal *Cryptococcus* species identification only became routinely accessible after 2018 and 2019, respectively. Liposomal amphotericin B was not available in our center until 2022, with only conventional deoxycholate amphotericin B used beforehand. Such temporal variations in diagnosis and treatment may bias our clinical outcomes. Furthermore, due to technological limitations, we were unable to perform quantitative cultures. Finally, this meta-analysis was limited by the relatively small number of included datasets and insufficient consideration of treatment regimen differences between Western countries and China, the statistical heterogeneity (*I*^2^ = 0, *P* = .52) may not adequately reflect the true clinical heterogeneity.

In this study, we confirm that the overall mortality rate of HIV-negative cryptococcosis patients is relatively low and mainly occurs within 2–10 weeks. Compared to Western countries, the characteristics of 2-week mortality rate show significant differences, primarily due to the differences in age distribution and proportion of high-risk populations. In the future management for HIV-negative cryptococcosis, it remains essential to fully consider these findings of the mortality characteristics.

## Supplementary Material

ofag410_Supplementary_Data

## Supplementary Data

Supplementary materials are available at *Open Forum Infectious Diseases* online. Consisting of data provided by the authors to benefit the reader, the posted materials are not copyedited and are the sole responsibility of the authors, so questions or comments should be addressed to the corresponding author.
