# Supplementary material for: Characteristics of Mortality in HIV-negative Cryptococcosis Patients: Analysis of a Cohort of 743 Patients
Source: Open Forum Infect Dis. 2026 Jul 14;13(7):ofag410. doi: 10.1093/ofid/ofag410 (PMC13373789; doi:10.1093/ofid/ofag410)
Supplement: ofag410_Supplementary_Data [file ofag410_supplementary_data.zip › Supplemental Material.docx]

**Supplemental Material**

**Contents**

[Supplementary Table 1. 2](#_Toc4095)

[Supplementary Table 2. 6](#_Toc18147)

[Supplementary Table 3. 7](#_Toc7256)

[Supplementary Figure 1 8](#_Toc15689)

[Supplementary Figure 2 9](#_Toc1911)

Definitions.......................................................................................................... 10

[Statistical Analysis 1](#_Toc12058)1

[Reference 16](#_Toc15903)

**Supplementary Table 1. Characteristics of the 743 HIV-negative patients with cryptococcosis (detailed).**

| **Characteristic** | **All patients**  **(N = 743)** | **Immune Condition** | | **P Value** |
| --- | --- | --- | --- | --- |
|  |  | **Patients with at least one known underlying immunocompromising condition(s) before cryptococcosis**  **(N = 304)** | **Patients with no identifiable immunocompromising condition**  **(N = 439)** |  |
| **A. Socio-demographic characteristics** |  |  |  |  |
| Male | 511 (68.8) | 192 (63.2) | 319 (72.7) | 0.01 |
| Age, y, median (IQR) | 47.0 (37.0, 59.0) | 47.5 (38.0, 59.0) | 47.0 (36.0, 59.0) | 0.66 |
| Rural residence (vs metropolitan) | 393 (52.9) | 154 (50.7) | 239 (54.4) | 0.35 |
| Smoking | 133 (17.9) | 58 (19.1) | 75 (17.1) | 0.55 |
| Alcohol consumption | 78 (10.5) | 33 (10.9) | 45 (10.3) | 0.89 |
| **B. Clinical characteristics** |  |  |  |  |
| Initial mRS score, mean ± SD | 1.0 (1.0, 2.0) | 1.0 (1.0, 2.0) | 1.0 (1.0, 2.5) | 0.35 |
| Initial GCS score <15 | 96 (12.9) | 39 (12.8) | 57 (13.0) | 1 |
| Site (s) of infection |  |  |  |  |
| CNS | 676 (91.0) | 279 (91.8) | 397 (90.4) | 0.62 |
| Lung | 78 (10.5) | 30 (9.9) | 48 (10.9) | 0.73 |
| Blood^a^, n = 289 | 34 (11.8) | 19 (15.3) | 15 (9.1) | 0.15 |
| Skin | 5 (0.7) | 2 (0.7) | 3 (0.7) | 1 |
| Bone | 4 (0.5) | 2 (0.7) | 2 (0.5) | 1 |
| Other (Soft tissue, adrenal gland and/or prostate) | 2 (0.3) | 0 (0.0) | 2 (0.5) | 0.52 |
| Disseminated | 51 (6.9) | 26 (8.6) | 25 (5.7) | 0.17 |
| Presenting signs/symptoms |  |  |  |  |
| No symptoms | 26 (3.5) | 11 (3.6) | 15 (3.4) | 1 |
| Fever ≥38°C | 307 (41.3) | 134 (44.1) | 173 (39.4) | 0.23 |
| Nausea/Vomiting | 327 (44.0) | 124 (40.8) | 203 (46.2) | 0.16 |
| Neurological features | 659 (88.7) | 270 (88.8) | 389 (88.6) | 1 |
| Headaches | 612 (82.4) | 249 (81.9) | 363 (82.7) | 0.86 |
| Dizziness | 146 (19.7) | 60 (19.7) | 86 (19.6) | 1 |
| Seizure | 4 (0.5) | 3 (1.0) | 1 (0.2) | 0.31 |
| Vision impairment | 161 (21.7) | 60 (19.7) | 101 (23.0) | 0.33 |
| Hearing impairment | 67 (9.0) | 30 (9.9) | 37 (8.4) | 0.59 |
| Altered mental status | 96 (12.9) | 39 (12.8) | 57 (13.0) | 1 |
| Other neurological symptoms/sign | 46 (6.2) | 23 (7.6) | 23 (5.2) | 0.25 |
| Respiratory symptoms (cough, sputum production, and/or dyspnea) | 42 (5.7) | 19 (6.2) | 23 (5.2) | 0.67 |
| Chest pain | 15 (2.0) | 5 (1.6) | 10 (2.3) | 0.61 |
| Skin lesion | 5 (0.7) | 2 (0.7) | 3 (0.7) | 1 |
| Lump pain | 2 (0.3) | 1 (0.3) | 1 (0.2) | 1 |
| Urinary system symptoms (Frequent urination, urgency, and/or painful urination) | 1 (0.1) | 0 (0.0) | 1 (0.2) | 1 |
| If symptomatic: time from onset of symptoms to diagnosis, d, median (IQR), n = 702 | 23.5 (14.0, 49.0) | 22.0 (12.0, 42.2) | 25.0 (14.2, 53.0) | 0.03 |
| **C. Diagnostic/Investigation results** |  |  |  |  |
| Cryptococcus species, n = 257 |  |  |  |  |
| *C. neoformans* | 239 (93.0) | 122 (97.6) | 117 (88.6) | 0.01 |
| *C. gattii* | 18 (7.0) | 3 (2.4) | 15 (11.4) | 0.01 |
| Baseline blood chemistry, median (range) |  |  |  |  |
| Aspartate aminotransferase, U/L, n = 713 | 20.0 (15.0, 29.0) | 21.0 (15.0, 29.8) | 19.0 (15.0, 29.0) | 0.37 |
| Alanine aminotransferase, U/L, n = 708 | 26.0 (16.0, 43.0) | 26.0 (15.2, 40.0) | 26.0 (16.0, 45.8) | 0.32 |
| Total protein, g/L, n = 706 | 65.6 (60.4, 70.7) | 63.4 (57.4, 68.9) | 66.7 (62.7, 72.3) | <0.01 |
| Albumin, g/L, n = 707 | 39.5 (35.8, 42.6) | 37.5 (33.3, 41.2) | 40.6 (37.5, 43.5) | <0.01 |
| Globulin, g/L, n = 706 | 26.0 (23.1, 29.2) | 25.2 (22.5, 28.5) | 26.4 (23.5, 29.6) | 0.01 |
| Total bilirubin, µmol/L, n = 617 | 9.7 (6.4, 14.3) | 10.2 (6.3, 15.7) | 9.6 (6.4, 13.9) | 0.25 |
| Direct bilirubin, µmol/L, n = 324 | 4.1 (2.7, 6.1) | 4.1 (2.7, 6.2) | 4.1 (2.8, 6.1) | 0.9 |
| Indirect bilirubin, µmol/L, n = 617 | 6.3 (3.9, 8.9) | 6.2 (3.9, 9.8) | 6.3 (4.0, 8.5) | 0.48 |
| Gamma-glutamyl transferase, U/L, n = 589 | 43.0 (27.0, 81.0) | 39.0 (25.0, 77.0) | 45.0 (29.0, 86.5) | 0.06 |
| Alkaline phosphatase, U/L, n = 588 | 75.0 (59.0, 97.0) | 69.0 (54.0, 90.0) | 79.0 (62.0, 100.0) | <0.01 |
| Potassium, mmol/L, n = 710 | 3.7 (3.3, 4.1) | 3.6 (3.3, 4.0) | 3.7 (3.3, 4.1) | 0.33 |
| Sodium, mmol/L, n = 710 | 137.0 (133.0, 140.1) | 137.0 (133.0, 140.0) | 136.6 (133.0, 140.3) | 0.83 |
| Chloride, mmol/L, n = 710 | 98.1 (94.5, 102.6) | 98.7 (95.1, 103.0) | 97.8 (94.1, 102.0) | 0.08 |
| Blood urea nitrogen, mmol/L, n = 365 | 4.7 (3.5, 6.3) | 4.7 (3.3, 6.0) | 4.7 (3.6, 6.4) | 0.37 |
| Creatinine, µmol/L, n = 715 | 67.0 (54.0, 85.0) | 68.0 (54.0, 88.0) | 66.0 (54.0, 83.0) | 0.29 |
| Uric acid, µmol/L, n = 639 | 251.0 (167.8, 340.5) | 235.5 (162.0, 347.0) | 261.4 (174.5, 335.0) | 0.5 |
| Total cholesterol, mmol/L, n = 571 | 4.3 (3.6, 5.2) | 4.3 (3.5, 5.1) | 4.3 (3.7, 5.2) | 0.41 |
| Triglycerides, mmol/L, n = 570 | 1.1 (0.8, 1.7) | 1.1 (0.8, 1.7) | 1.2 (0.8, 1.7) | 0.44 |
| Low-density lipoprotein, mmol/L, n = 571 | 2.7 (2.1, 3.4) | 2.7 (2.1, 3.4) | 2.7 (2.2, 3.4) | 0.89 |
| C-reactive protein, mg/L, n = 381 | 6.9 (2.2, 20.6) | 8.6 (2.5, 23.5) | 6.0 (2.1, 18.4) | 0.12 |
| Baseline bloodwork, median (range) |  |  |  |  |
| White blood cell, 10^9/L, n = 722 | 8.4 (6.2, 11.6) | 7.7 (5.7, 10.6) | 8.9 (6.6, 12.0) | <0.01 |
| Hemoglobin, g/L, n = 725 | 125.0 (110.0, 139.0) | 121.5 (105.0, 136.0) | 127.0 (114.0, 141.0) | <0.01 |
| Platelet, g/L, n = 726 | 247.0 (191.2, 305.0) | 226.0 (157.0, 277.0) | 259.0 (219.0, 319.0) | <0.01 |
| Neutrophil , 10^9/L, n = 726 | 1.2 (0.8, 1.7) | 1.1 (0.6, 1.6) | 1.3 (0.9, 1.7) | <0.01 |
| Lymphocyte , 10^9/L, n = 726 | 6.4 (4.4, 9.4) | 5.9 (4.1, 8.6) | 6.8 (4.6, 9.6) | <0.01 |
| Monocyte , 10^9/L, n = 726 | 0.6 (0.4, 0.8) | 0.6 (0.3, 0.8) | 0.6 (0.4, 0.8) | 0.16 |
| Reticulocyte , 10^9/L, n = 333 | 63.9 (47.8, 87.4) | 64.6 (51.1, 87.2) | 63.8 (45.8, 87.4) | 0.27 |
| CSF findings (for patients with CNS cryptococcosis) |  |  |  |  |
| Opening pressure, cmH2O, n = 652 |  |  |  |  |
| <20 | 216 (33.1) | 95 (35.1) | 121 (31.8) | 0.28 |
| 20–30 | 279 (42.8) | 106 (39.1) | 173 (45.4) | 0.28 |
| >30 | 157 (24.1) | 70 (25.8) | 87 (22.8) | 0.28 |
| CSF cryptococcus count, count/mL, median (IQR), n = 567 | 1320.0 (25.0, 12297.0) | 948.5 (22.0, 12682.0) | 1560.0 (36.0, 11246.0) | 0.66 |
| White blood cell, 10^6/L, median (IQR), n = 668 | 82.0 (32.0, 174.0) | 78.0 (28.0, 172.0) | 87.0 (34.0, 174.5) | 0.27 |
| Glucose, mmol/L, median (IQR), n = 664 | 0.8 (0.5, 1.2) | 0.7 (0.5, 1.2) | 0.8 (0.5, 1.3) | 0.12 |
| Protein, g/L, median (IQR), n = 664 | 1.8 (0.8, 2.7) | 2.1 (0.9, 2.9) | 1.7 (0.8, 2.5) | <0.01 |
| Chloride, mmol/L, median (IQR), n = 664 | 117.5 (112.1, 121.9) | 117.5 (112.2, 121.9) | 117.5 (111.9, 121.9) | 0.88 |
| Positive India Ink Staining, n = 674 | 591 (87.7) | 240 (86.3) | 351 (88.6) | 0.44 |
| Positive culture, n = 665 | 424 (63.8) | 196 (71.0) | 228 (58.6) | <0.01 |
| Positive CSF CrAg, n = 260 | 258 (99.2) | 126 (99.2) | 132 (99.2) | 1 |
| Positive serum CrAg, n = 189 | 180 (95.2) | 90 (94.7) | 90 (95.7) | 1 |
| Positive blood cultures, n = 289 | 34 (11.8) | 19 (15.3) | 15 (9.1) | 0.15 |
| Brain imaging findings (for patients with CNS cryptococcosis), n = 588 |  |  |  |  |
| Cryptococcoma(s) | 88 (15.0) | 34 (14.1) | 54 (15.6) | 0.71 |
| Gelatinous pseudocystic lesion | 10 (1.7) | 2 (0.8) | 8 (2.3) | 0.21 |
| Meningeal enhancement | 285 (48.5) | 113 (46.9) | 172 (49.6) | 0.58 |
| Hydrocephalus | 106 (18.0) | 42 (17.4) | 64 (18.4) | 0.84 |
| No characteristic lesions reported | 226 (38.4) | 99 (41.1) | 127 (36.6) | 0.31 |
| **D. Treatment and treatment response^b^** |  |  |  |  |
| PIIRS/IRIS-like reconstitution syndrome (for patients with CNS cryptococcosis), n = 676 | 70 (10.4) | 21 (7.5) | 49 (12.3) | 0.06 |
| Persistent infection, n = 227 | 15 (6.6) | 11 (11.0) | 4 (3.1) | 0.03 |
| Microbiological relapse, n = 227 | 1 (0.4) | 1 (1.0) | 0 (0.0) | 0.44 |
| **E. Outcomes** |  |  |  |  |
| All-cause mortality, 95% CI |  |  |  |  |
| 2 week | 1.4 (0.5, 2.19) | 1.0 (0.0, 2.1) | 1.59 (0.4, 2.8) | 0.48 |
| 10 week | 8.4(6.3-10.4) | 10.53 (7.5, 14.9) | 6.2 (4.1, 8.8) | 0.03 |
| 1 year | 12.2(9.7-14.7) | 13.5 (10.4, 19.0) | 9.8 (7.5, 13.6) | 0.1 |

Note: Data are n (%) unless otherwise indicated. a: means the result of positive Cryptococcus in blood culture. b: Specific medications and surgical treatments are detailed in Supplementary Table 4. CrAg testing was implemented at our institution in 2018.

Abbreviations: IQR, interquartile range; mRS, modified Rankin Scale; GCS, Glasgow Coma Scale; CNS, central nervous system; CSF, cerebrospinal fluid; CrAg, cryptococcal antigen; CI, confidence interval; HIV, human immunodeficiency virus; PIIRS, paradoxical immune inflammatory response syndrome; IRIS, immune reconstitution inflammatory syndrome.

**Supplementary Table 1. Immune condition of the 743 HIV-negative patients with cryptococcosis.**

| **Immune condition** | **All Patients (N = 743)** |
| --- | --- |
| **Patients with no identifiable immunocompromising condition before cryptococcosis** | 439 (59.1) |
| **Patients with at least one known underlying immunocompromising condition(s) before cryptococcosis ^a^** | 304 (40.9) |
| **Viral hepatitis** | 133 (43.8) |
| **Immunosuppressive drugs** | 103 (33.9) |
| **Glucocorticoid therapy** | 80 (26.3) |
| **Cytotoxic chemotherapy** | 17 (5.6) |
| **Calcineurin/mTOR inhibitors** | 16 (5.3) |
| **Antimetabolites** | 16 (5.3) |
| **Immunomodulator** | 21 (6.9) |
| **Other** | 8 (2.6) |
| **Diabetes** | 84 (27.6) |
| **Systemic lupus erythematosus** | 38 (12.5) |
| **Immune nephropathy** | 38 (12.5) |
| **Cancer** | 26 (8.6) |
| **Rheumatoid arthritis** | 15 (4.9) |
| **Solid organ transplantation** | 13 (4.3) |
| **Idiopathic thrombocytopenic purpura** | 7 (2.3) |
| **Autoimmune hemolytic anemia** | 5 (1.6) |
| **Autoimmune hepatitis** | 5 (1.6) |
| **Sarcoidosis.** | 4 (1.3) |
| **Dermatomyositis** | 4 (1.3) |
| **Vasculitis** | 2 (0.7) |
| **Inflammatory bowel disease** | 2 (0.7) |
| **Wegener's granulomatosis** | 1 (0.3) |
| **Behçet’s disease** | 1 (0.3) |
| **Optic neuromyelitis** | 1 (0.3) |

Note: a, one patient may have multiple comorbidities. HIV, human immunodeficiency virus.

**Supplementary Table 2. Univariate and multivariate Cox regression analysis of mortality risk factors in cryptococcosis patients for all or CNS patients.**

| **Parameters** | **Univariate analysis** | | **Multivariable analysis** | |
| --- | --- | --- | --- | --- |
|  | **Hazard ratio (95% CI)** | **P value** | **Hazard ratio (95% CI)** | **P value** |
| **All patients (N=743)** |  |  |  |  |
| Age | 2.966 [1.868,4.712] | <0.001 | 1.962 [1.204,3.198] | 0.009 |
| Initial mRS score | 1.724 [1.515,1.961] | <0.001 | 1.606 [1.347,1.914] | 0.000 |
| Fever≥38°C | 2.790 [1.785,4.360] | <0.001 | 1.596 [0.995,2.560] | 0.057 |
| Altered mental status | 5.431 [3.511,8.401] | <0.001 | 1.956 [1.099,3.481] | 0.026 |
| Number of symptoms | 1.264 [1.029,1.552] | 0.025 | 0.886 [0.680,1.153] | 0.370 |
| Total protein | 0.601 [0.377,0.959] | 0.031 | 1.548 [0.816,2.938] | 0.189 |
| Albumin | 0.367 [0.235,0.573] | <0.001 | 0.436 [0.237,0.802] | 0.010 |
| Total bilirubin | 2.740 [1.497,5.016] | 0.001 | 2.145 [1.113,4.132] | 0.030 |
| Creatinine | 2.170 [1.256,3.750] | 0.004 | 2.408 [1.485,3.903] | 0.001 |
| White blood cell count | 3.233 [2.066,5.061] | <0.001 | 1.843 [1.156,2.939] | 0.013 |
| **CNS patients (N=676)** |  |  |  |  |
| Age | 2.966 [1.865,4.716] | <0.001 | 2.100 [1.282,3.439] | 0.004 |
| Initial mRS score | 1.680 [1.472,1.917] | <0.001 | 1.555 [1.312,1.843] | <0.001 |
| Fever≥38°C | 2.517 [1.603,3.953] | <0.001 | 1.516 [0.940,2.445] | 0.093 |
| Headaches | 0.393 [0.230,0.669] | <0.001 | 0.752 [0.422,1.340] | 0.337 |
| Altered mental status | 4.938 [3.186,7.652] | <0.001 | 1.884 [1.065,3.331] | 0.033 |
| Positive India ink staining | 2.869 [1.050,7.836] | 0.031 | 3.140 [1.095,9.003] | 0.037 |
| Total protein | 0.626 [0.392,0.999] | 0.048 | 1.575 [0.862,2.879] | 0.144 |
| Albumin | 0.402 [0.256,0.630] | <0.001 | 0.410 [0.219,0.768] | 0.007 |
| Total bilirubin | 2.599 [1.419,4.763] | 0.001 | 1.859 [0.978,3.534] | 0.065 |
| Creatinine | 1.945 [1.109,3.409] | 0.018 | 2.319 [1.401,3.840] | 0.002 |
| White blood cell count | 2.896 [1.847,4.540] | <0.001 | 1.725 [1.063,2.798] | 0.032 |

Abbreviations: mRS, modified Rankin scale; CI, 95% Confidence Interval

**Supplementary Table 3. Comparison of baseline age characteristics among risk groups in studies on HIV-negative cryptococcosis patients**

| Characteristic | Age | | | |
| --- | --- | --- | --- | --- |
|  | Australia and New Zealand, mean ± SD | France  , median（ IQR） | Eastern China  , median（ IQR） | Our study  , median  （ IQR） |
|  |  |  |  |  |
|  |  |  |  |  |
| All patients | NA | 61 (48-72) | 47 (35-60) | 47.0 (37.0,-59.0) |
| Underlying risks |  |  |  |  |
| Malignancy | 67 ± 13 | 68 (58-75) | NA | 52（48.25-62.5） |
| Solid Organ Transplant | 54.9 ± 12.2 | 58 (48-66) | NA | 49（35.5-59） |
| Other immunocompromising condition | 61.2 ± 15 | 57 (44-71.5) | NA | 47（37-58） |
| No identified condition | 54 ± 15.3 | 52 (35-70) | NA | 47（36-59） |

Abbreviations: NA, Not Available; IQR, Interquartile Range; SD, Standard Deviation

**Supplementary Figure 1**

**
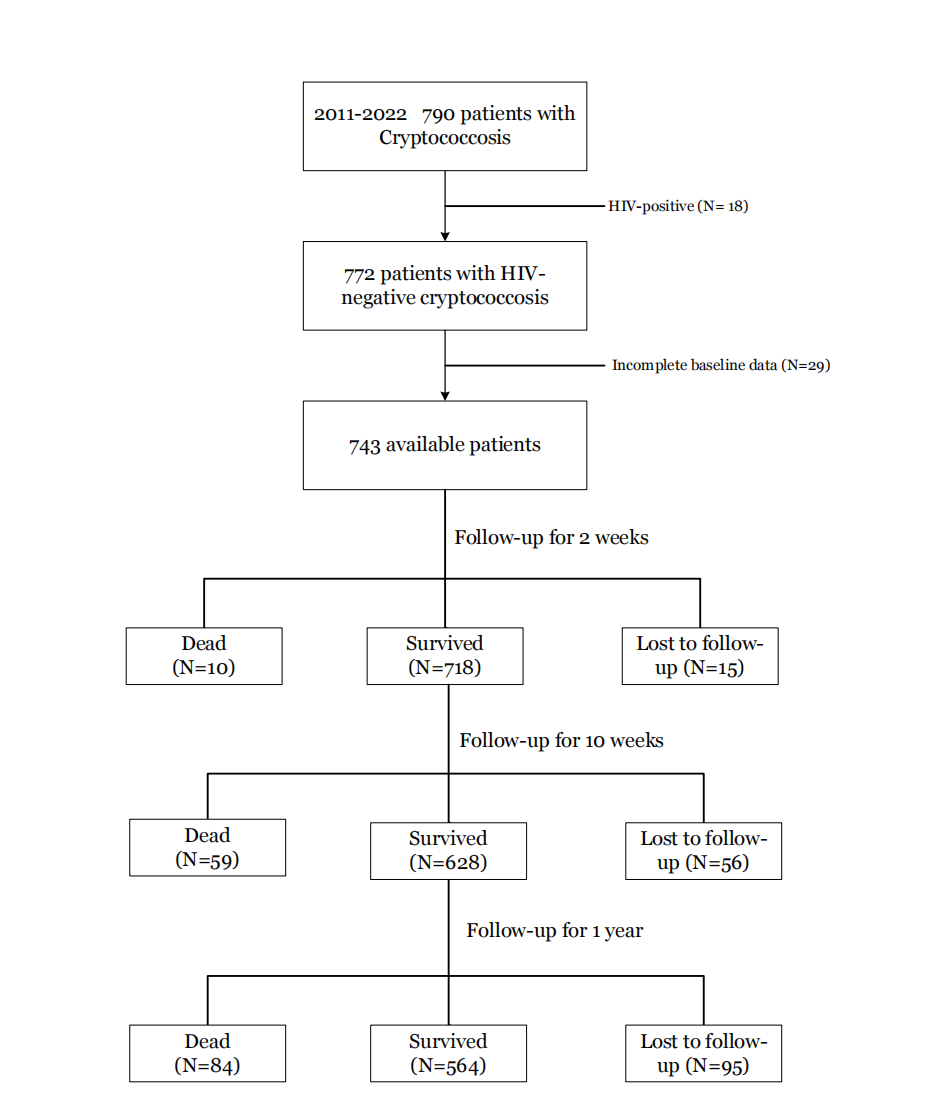
**

**Supplementary Figure
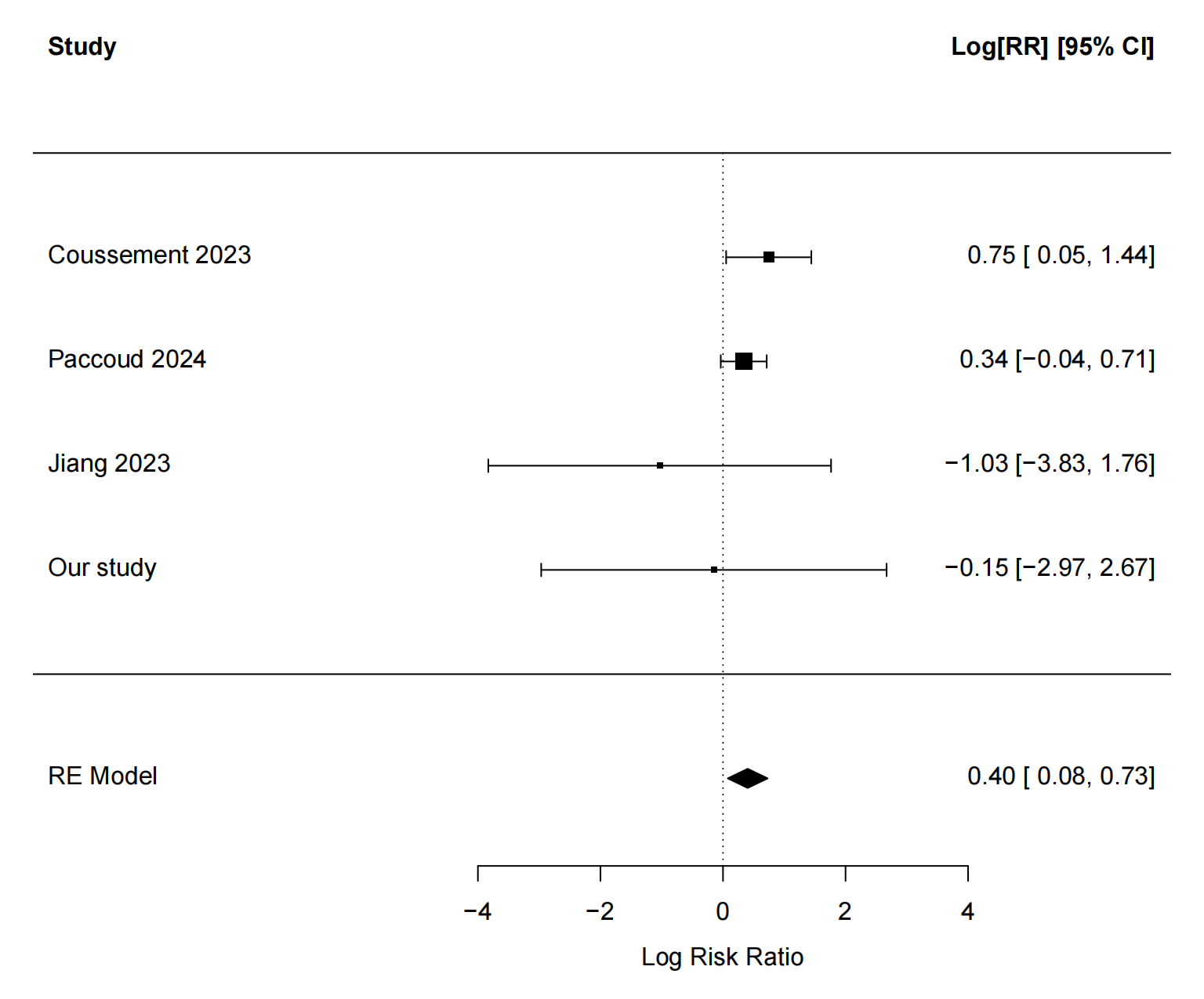
2**

**Definitions**

The study’s inclusion and exclusion criteria are as follows: I. Inclusion Criteria: 1. Definitive diagnosis of cryptococcosis in accordance with the 2019 EORTC/MSG-ERC consensus definition via the aforementioned diagnostic methods. The definition of cryptococcosis was based on the consensus definition of invasive fungal diseases by the European Organization for Research and Treatment of Cancer (EORTC) and the Mycoses Study Group Education and Research Consortium (MSG-ERC), revised and updated in 2019 [1]. Confirmed cases were those diagnosed by microscopy, culture, antigen, and tissue nucleic acid detection . Disseminated ced as infection involving at least two non-contiguous sites. As most of the cryptococcosis patients in this study did not undergo blood culture testing, we were unable to determine whether they had fungemia. Therefore, all patients with CNS cryptococcal infections, regardless of the presence of fungemia, were classified as CNS cryptococcosis. 2. Negative HIV test result; 3. Admission time from 2011 to 2022; II. Exclusion Criteria: 1. Incomplete baseline data; 2. Positive HIV test result. Persistent infection was defined as a persistently positive cryptococcal culture in CSF for 4 weeks after starting antifungal therapy . Microbiologicaas recurrence of symptoms, with recovery of viable organisms from previously sterile CSF . Paradoxical imonse syndrome (PIIRS) is defined as paradoxical clinical and/or radiological deterioration in CM patients who were previously healthy and without immunological abnormalities, occurring during effective antifungal treatment due to a strong immune response, with consistently negative fungal cultures . Altered mental a Glasgow coma score (GCS) of < 15. HIV-negative patients are categorized into two major classes of underlying immunocompromising conditions and one of four primary potential risk groups in the following order: 1. Patients with at least one known underlying immunocompromising condition prior to cryptococcosis: (1) Solid organ transplant (SOT), (2) Malignancy, (3) Other immunocompromising condition. 2. Patients without known underlying immunocompromising conditions: (4) No documented risk factors.

**Statistical Analysis**

**Epidemiology Analysis:**

The Shapiro-Wilk test is used to assess whether continuous variables are normally distributed. Continuous variables that are not normally distributed are presented as the median with interquartile range (IQR), while normally distributed continuous variables are reported as the mean with standard error. To compare these variables across different groups, the Kruskal-Wallis rank sum test is applied for non-normally distributed data, and one-way ANOVA is used for normally distributed data.

Categorical variables are generally compared using the Chi-square test with a large sample approximation. If any group has fewer than 5 observations, Fisher’s exact test is conducted instead.

**Mortality Analysis**

All-cause mortality at 2 weeks, 10 weeks, 6 months, and 1 year and the corresponding 95% confidence intervals (CIs) are estimated using Kaplan-Meier estimates.

Comparisons of all-cause mortality between groups were performed using log-rank tests. The Cox proportional hazards model, with group indicators as predictors, was used to derive hazard ratios (HRs) and 95% CIs. Kaplan-Meier plots were constructed to visualize survival differences.

The group indicators include: (1) Immune status, (2) Cryptococcus diagnosis, (3) Cerebrospinal fluid culture, (4) Drug treatment, (5) Blood culture: conducted or not, (6) Blood culture: positive or negative, and (7) Ventriculoperitoneal shunt.

**Risk Factor Analysis**

The study outcomes are defined as all-cause mortality at 1-year, 10-week and 2-week. Cox proportional hazards regression models were used to identify risk factors for these outcomes. Risk factor analysis was performed on both the full cohort and the subset of patients with central nervous system (CNS) infections, considering different potential risk factors.

Risk factor analysis was performed on both the full cohort and the subset of patients with central nervous system (CNS) infections, considering different potential risk factors.

The model integrated a predefined set of clinically relevant variables for comprehensive analysis. 1. For all cryptococcal patients, the variables included gender (male or female), age (≥50 years or <50 years), immune status (immunocompromised or normal), initial modified Rankin Scale (mRS) score, fever (≥38°C or <38°C), altered mental status (Glasgow Coma Scale score <15), number of comorbidities (14 symptoms), time from symptom onset to diagnosis (≥30 days or <30 days), alanine aminotransferase (ALT, ≥50 U/L or <50 U/L), total bilirubin (Tbil, ≥24 μmol/L or <24 μmol/L), gamma-glutamyl transferase (GGT, ≥60 U/L or <60 U/L), total protein (TPROT, ≥60 g/L or <60 g/L), albumin (ALB, ≥35 g/L or <35 g/L), creatinine (CRE, ≥110 μmol/L or <110 μmol/L), uric acid (UA, ≥420 μmol/L or <420 μmol/L), C-reactive protein (CRP, ≥10 mg/L or <10 mg/L), potassium (K, ≥3.5 mmol/L or <3.5 mmol/L), sodium (Na, ≥135 mmol/L or <135 mmol/L), white blood cell count (WBC, ≥10×10^9/L or <10×10^9/L), hemoglobin (HGB, ≥110 g/L or <110 g/L), platelet count (Plt, ≥100×10^9/L or <100×10^9/L), and neutrophil count (NEUT#, ≥1.5×10^9/L or <1.5×10^9/L).

For patients with central nervous system (CNS) cryptococcal infection, additional variables were considered, including gender (male or female), age (≥50 years or <50 years), immune status (immunocompromised or normal), initial mRS score, fever (≥38°C or <38°C), headache, altered mental status (Glasgow Coma Scale score <15), visual impairment, hearing impairment, number of symptoms (14 symptoms), time from symptom onset to diagnosis (≥30 days or <30 days), alanine aminotransferase (ALT, ≥50 U/L or <50 U/L), total bilirubin (Tbil, ≥24 μmol/L or <24 μmol/L), gamma-glutamyl transferase (GGT, ≥60 U/L or <60 U/L), total protein (TPROT, ≥60 g/L or <60 g/L), albumin (ALB, ≥35 g/L or <35 g/L), creatinine (CRE, ≥110 μmol/L or <110 μmol/L), uric acid (UA, ≥420 μmol/L or <420 μmol/L), C-reactive protein (CRP, ≥10 mg/L or <10 mg/L), potassium (K, ≥3.5 mmol/L or <3.5 mmol/L), and sodium (Na, ≥135 mmol/L or <135 mmol/L).

These variables were incorporated into the model to thoroughly evaluate potential associations between clinical characteristics and study outcomes.

These risk factors were predefined baseline characteristics based on clinical knowledge. Characteristics available for at least 80% of participants were included. The baseline characteristics are shown in Table 1.

For each baseline characteristic, if the univariable analysis showed a significant association with the outcome (P value <0.05), the characteristic was included in a subsequent multivariable model.

The corresponding records for these characteristics at 2 weeks after the baseline are included in the multivariable model if exists. Subjects that miss 50% characteristics are excluded in the multivariable analysis. Missing values in the multivariable model were handled using predictive mean matching within the framework of multivariate imputation by chained equations. The final estimates of hazard ratios (HRs) and CIs for risk factors were pooled results from five imputed datasets using Rubin’s rules.

(Results: Sample interpretation)

[1] Cox proportional hazard model with time-dependent: coefficient interpret example

Age: coefficient=0.577, hazard ratio = 1.781

The coefficient of 0.577 for 'Age' in the time-dependent Cox model suggests that, on average, each additional year of age increases the hazard of the event by about 78.1%, assuming all other factors remain constant.

The coefficient itself suggests that the effect of age on the hazard remains consistent over time.

[2] Cox proportional hazard model with time-dependent: compared with model with constant variables

There’re almost the same with the only difference is “positive india ink staining”

The identification of similar risk factors by both the Cox proportional hazards model with constant baseline variables and the model incorporating a second measurement at 2 weeks suggests that the baseline characteristics are robust and reliable predictors of the outcome. This consistency indicates that the baseline data alone provides a comprehensive risk profile, with early changes at 2 weeks not significantly altering the risk factors. Thus, the initial assessment appears sufficient for identifying key risk factors, implying that the impact of these factors remains stable over time.

**Sensitivity Analysis**

A sensitivity analysis was conducted to assess whether including the covariate of blood culture results (negative or positive) would influence the outcomes of the risk factor analysis in patients who had a blood culture performed.

**Meta-analysis**

A systematic search was conducted in the PubMed database for literature published up to December 31, 2024. Pubmed database retrieval expression: (Cryptococcus OR Cryptococcosis OR "cryptococcal infection" OR "Cryptococcus neoformans" OR "Cryptococcus gattii") AND ("HIV negative" OR "HIV seronegative" OR "non-HIV" OR "without HIV") AND (mortality OR "death rate" OR fatality OR "lethal outcome") NOT ("HIV positive" OR "HIV-infected").

Study Selection and Data Extraction

Two investigators independently screened the literature, and discrepancies were resolved by a third investigator.

Inclusion criteria:

1. Population: HIV-negative patients diagnosed with cryptococcal infection;

2. Study design: Cohort studies, case series, or clinical trials reporting all-cause mortality within 14 days;

3. Risk stratification: Clear reporting of grouped data for solid organ transplant (SOT), malignancy, other immunocompromising conditions, or no documented risk factors.

4. Publication Date: Studies must have been published on or after January 1, 2014.

5. Studies were excluded if they mixed HIV-positive and HIV-negative populations without separate reporting, were case reports, or had duplicated data.

Results

A total of 104 records were initially identified. After full-text screening, 3 studies were ultimately included.

**Reference**

1. Donnelly JP, Chen SC, Kauffman CA, Steinbach WJ, Baddley JW, Verweij PE, Clancy CJ, Wingard JR, Lockhart SR, Groll AH, Sorrell TC, Bassetti M, Akan H, Alexander BD, Andes D, Azoulay E, Bialek R, Bradsher RW, Bretagne S, Calandra T, Caliendo AM, Castagnola E, Cruciani M, Cuenca-Estrella M, Decker CF, Desai SR, Fisher B, Harrison T, Heussel CP, Jensen HE, Kibbler CC, Kontoyiannis DP, Kullberg BJ, Lagrou K, Lamoth F, Lehrnbecher T, Loeffler J, Lortholary O, Maertens J, Marchetti O, Marr KA, Masur H, Meis JF, Morrisey CO, Nucci M, Ostrosky-Zeichner L, Pagano L, Patterson TF, Perfect JR, Racil Z, Roilides E, Ruhnke M, Prokop CS, Shoham S, Slavin MA, Stevens DA, Thompson GR, Vazquez JA, Viscoli C, Walsh TJ, Warris A, Wheat LJ, White PL, Zaoutis TE, Pappas PG. Revision and Update of the Consensus Definitions of Invasive Fungal Disease From the European Organization for Research and Treatment of Cancer and the Mycoses Study Group Education and Research Consortium. Clin Infect Dis. 2020 Sep 12; 71(6): 1367-1376.

2. Perfect JR, Dismukes WE, Dromer F, Goldman DL, Graybill JR, Hamill RJ, Harrison TS, Larsen RA, Lortholary O, Nguyen MH, Pappas PG, Powderly WG, Singh N, Sobel JD, Sorrell TC. Clinical practice guidelines for the management of cryptococcal disease: 2010 update by the infectious diseases society of america. Clin Infect Dis. 2010 Feb 1; 50(3): 291-322.

3. Williamson PR, Jarvis JN, Panackal AA, Fisher MC, Molloy SF, Loyse A, Harrison TS. Cryptococcal meningitis: epidemiology, immunology, diagnosis and therapy. Nat Rev Neurol. 2017 Jan; 13(1): 13-24.

4. Williamson PR. Post-infectious inflammatory response syndrome (PIIRS): Dissociation of T-cell-macrophage signaling in previously healthy individuals with cryptococcal fungal meningoencephalitis. Macrophage (Houst). 2015; 2: e1078.
