# Supplementary material for: Characteristics of Mortality in HIV-negative Cryptococcosis Patients: Analysis of a Cohort of 743 Patients
Source: Open Forum Infect Dis. 2026 Jul 14;13(7):ofag410. doi: 10.1093/ofid/ofag410 (PMC13373789; doi:10.1093/ofid/ofag410)
Supplement: ofag410_Supplementary_Data [file ofag410_supplementary_data.zip › Supplementary_Legends.docx]

**Supplementary Materials**

**Supplemental Figure 1. The flowchart for inclusion of patients**

**Supplemental Figure 2. Forest plot of the meta-analysis of association between immunocompromise conditions and mortality.** The summarized log risk ratio is estimated by a random-effect meta-analysis model.
